# Application of Tissue Aspirate Parathyroid Hormone Assay for Imaging Suspicious Neck Lesions in Patients with Complicated Recurrent or Persistent Renal Hyperparathyroidism

**DOI:** 10.3390/jcm10020329

**Published:** 2021-01-18

**Authors:** Chien-Ling Hung, Yu-Chen Hsu, Shih-Ming Huang, Chung-Jye Hung

**Affiliations:** 1 Department of Surgery, Tainan Sin Lau Hospital, Tainan 704302, Taiwan; olen1981@gmail.com; 2 Division of General Surgery, Department of Surgery, National Cheng Kung University Hospital, College of Medicine, National Cheng Kung University, Tainan 704302, Taiwan; smhuang@mail.ncku.edu.tw; 3 Department of Surgery, Chia-Yi Christian Hospital, Chia-Yi 600566, Taiwan; seetowhat@yahoo.com.tw; 4 Asian International Thyroid Center, Chang Bing Show Chwan Memorial Hospital, Changhua 505029, Taiwan

**Keywords:** tissue aspirate parathyroid hormone assay, recurrent renal hyperparathyroidism, persistent renal hyperparathyroidism, parathyroid sonography, parathyroid scintigraphy

## Abstract

Background: Comprehensive pre-reoperative localization is essential in complicated persistent or recurrent renal hyperparathyroidism. The widely used imaging studies sometimes lead to ambiguous results. Our study aimed to clarify the role of tissue aspirate parathyroid hormone (PTH) assay with a new positive assay definition for imaging suspicious neck lesions in these challenging scenarios. Methods: All patients with complicated recurrent or persistent renal hyperparathyroidism underwent parathyroid sonography and scintigraphy. Echo-guided tissue aspirate PTH assay was performed in suspicious lesions revealed by localization imaging studies. The tissue aspirate PTH level was determined by an immunoradiometric assay. We proposed a newly-developed definition for positive assay as a washout level higher than one-thirtieth of the serum PTH level obtained at the same time. The final diagnosis after re-operation was confirmed by the pathologists. Results: In total, 50 tissue aspirate PTH assays were performed in 32 patients with imaging suspicious neck lesions, including discrepant results between scintigraphy and sonography in 47 lesions (94%), unusual locations in 19 lesions (38%), multiple foci in 28 lesions (56%), and locations over previously explored areas in 31 lesions (62%). Among 39 assay-positive lesions, 13 lesions (33.3%) were not identified by parathyroid scintigraphy, and 28 lesions (71.8%) had uncertain parathyroid sonography findings. The final pathology in patients who underwent re-operative surgery proved the tissue aspirate PTH assays had a 100% positive predictive value. Conclusions: Our findings suggest tissue aspirate PTH assay with this new positive assay definition is beneficial to clarify the nature of imaging suspicious lesions in patients with complicated persistent or recurrent renal hyperparathyroidism.

## 1. Introduction

Renal hyperparathyroidism is a common complication of chronic kidney disease and renal failure that can be further classified into secondary hyperparathyroidism or tertiary hyperparathyroidism according to the serum calcium level and the underlying mechanism of elevated parathyroid hormone (PTH) [1]. Improving medical treatment with vitamin D analogs, phosphate binders, and calcimimetic drugs expands the treatment options for these patients, but surgical treatment with parathyroidectomy remains necessary for many patients. The management of persistent or recurrent renal hyperparathyroidism is especially challenging for endocrine surgeons. Around 2.7–11.5% of patients needed reoperation due to persistence or recurrence after the primary operation [2,3,4]. However, such procedures are difficult and have a high complication rate due to adhesion, dense scar tissue, distortion of anatomic tissue, and loss of the normal plane [5]. It is generally agreed that accurate pre-reoperative localization is the cornerstone to reduce surgical risk and avoid negative neck exploration.

Parathyroid scintigraphy and sonography are the most widely used pre-reoperative localization methods. However, their sensitivity and accuracy are unsatisfactory and vary in different studies [6,7,8,9]. The accuracy of parathyroid scintigraphy is highly dependent on the size and functional status of the parathyroid glands, and thyroid lesions can lead to misinterpretations of the results [10,11]. The reported success rates of the pre-operative localization of parathyroid scintigraphy for reoperative renal hyperparathyroidism range from 71% to 93% in some small-sized studies [2,8,12], but the data remain scarce for patients with secondary or tertiary hyperparathyroidism [13]. Parathyroid sonography depends on the technical ability of the operator [14], and it is not uncommon for the findings to confuse thyroid nodules or lymph nodes. The results of parathyroid sonography for pre-reoperative localization in persistent or recurrent renal hyperparathyroidism are unsatisfactory, with a 50% sensitivity rate and a 16.7% false-positive rate shown in different studies [7,15]. Moreover, the interpretation can be complicated if there are uncertain or even discrepant results between these two localization studies. Therefore, another adjuvant tool to further clarify the results of localization studies is clearly needed.

The tissue aspirate parathyroid hormone (PTH) assay was first introduced in patients with primary hyperparathyroidism in 1983 by Doppman [16], and it is now also applied in patients with persistent or recurrent primary hyperparathyroidism featuring high sensitivity and specificity [17,18,19]. Nevertheless, no previous studies have reported the application of tissue aspirate PTH assays in patients with persistent or recurrent renal hyperparathyroidism. Therefore, in this study, we aimed to investigate the role of the tissue aspirate PTH assay with a new proposed positive assay definition for suspicious neck lesions for imaging in patients with complicated recurrent or persistent renal hyperparathyroidism.

## 2. Materials and Methods

### 2.1. Study Population

Patients with recurrent or persistent renal hyperparathyroidism who underwent localization studies between September 1995 and December 2014 at National Cheng Kung University Hospital (NCKUH) were enrolled in the study. Persistent renal hyperparathyroidism was defined as a PTH level higher than the upper normal range after the first operation [3]. Recurrent renal hyperparathyroidism was defined by a PTH level that dropped to or below a normal range after the first operation and rose above the normal range after 6 months [3]. All patients underwent localization studies including parathyroid sonography (neck and graft site) and parathyroid scintigraphy (neck, mediastinum, and graft site). An echo-guided tissue aspirate PTH assay was performed in each patient with suspicious or discrepant lesions revealed by the imaging results, either through sonography or scintigraphy. Informed consent was obtained from each patient before the procedure. The study was approved by the Institutional Ethics Review Board of NCKUH (A-ER-108-167).

### 2.2. Tissue Aspirate PTH Assay

Echo-guided tissue aspiration was performed by a single surgeon (C.J.H). The patients were asked to lie on a bed with their necks extended. After sterile preparation, the operator performed tissue aspiration with the dominant hand and held the sonography probe for localization with the other hand. A 23-gauge needle was inserted into the suspicious neck lesion under ultrasound guidance. After insertion, the operator fixed the needle and rapidly oscillated the piston 5~8 times. The aspirate was then washed out with 6 mL normal saline and sent for PTH measurement, using an immunoradiometric assay for intact PTH (Cisbio Bioassays, Codolet, France. normal range of 10–65 pg/mL), which was then compared with the serum PTH level measured before the procedure. The positive result was defined as a tissue aspirate PTH level higher than one-thirtieth of the serum PTH level. The patients were able to leave the clinic after compression of the aspiration site for 30 min. Parathyroidectomy was suggested in assay-positive cases. 

### 2.3. Data Analysis

**Figure 1:**
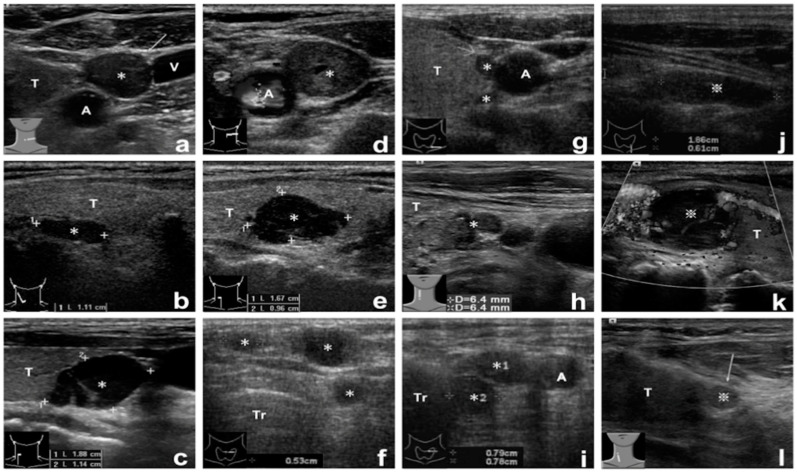
Images of positive and suspicious parathyroid sonography lesions. (**a**–**c**) Case 1, 2, 6, with positive sonographic lesions. (**d**) Case 5, with suspicious left upper neck lesion. (**e**) Case 20, with suspicious intrathyroid lesion. (**f**) Case 24, with suspicious multiple subcutaneous lesions. (**g**) Case 25, with suspicious dumbbell shape lesion. (**h**) Case 8, with suspicious infrathyroid lobulated lesion. (**i**) Case 26-1, with suspicious multiple paratracheal lesions. (**j**) Case 14, with suspicious intrathymus lesion (false assay-negative). (**k**) Case 31, with suspicious intrathyroid lesion. (**l**) Case 26-2, with suspicious infrathyroid small lesion. ✻ Positive tissue aspirate PTH assay lesions. ※ Negative tissue aspirate PTH assay lesions. Demographic data of enrolled patients are listed in Table S1. Abbreviations: A, common carotid artery; V, internal jugular vein; T, thyroid; Tr, trachea.

The patients received follow-ups until the end of June 2017. All data on demographics, localization, tissue aspirate PTH assay results, reoperative findings and procedures, pathological findings, and post-reoperative course were recorded and analyzed. The results of parathyroid sonography were classified as either suspicious or positive, and the results of parathyroid scintigraphy were classified as negative, suspicious, or positive. The suspicious parathyroid sonographic results were defined as neck lesions with unusual locations or atypical sonographic images including shape, size, and number (Figure 1). The results of parathyroid scintigraphy were determined with the agreement of both the surgeon and nuclear medicine specialist. The final diagnosis after reoperation was confirmed by the pathologists. The accuracy of the tissue aspirate PTH assays was calculated according to the reoperative results. 

### 2.4. Statistical Analysis

Continuous variables are expressed as the median and range unless stated otherwise. Categorical variables were expressed as numbers and percentages. Statistical significance was assessed by a Mann–Whitney U-test for continuous variables, and by a chi-square test and Fisher’s exact test for categorical variables. All analyses were performed using the SPSS software (SPSS Statistics for Windows, Version 17.0, SPSS Inc., Chicago, IL, USA). A *p* value < 0.05 was considered to be statistically significant. 

## 3. Results

### 3.1. Patient Demographics

In total, 115 patients with recurrent or persistent renal hyperparathyroidism underwent localization studies during the study period, and 50 tissue aspirate PTH assays were performed in 32 patients. The demographic data of the patients receiving tissue aspirate PTH assays are shown in Table S1. The mean age of these patients was 56.6 ± 7.6 years with female predominance (78.1%). Nine (28.1%) patients received their initial operation at another hospital, and eight patients (25%) underwent more than one operation before the tissue aspirate PTH assay. Four or more parathyroid glands were removed during a previous surgery in at least 21 patients (65.6%). Twenty-four patients were classified as having persistent renal hyperparathyroidism and eight as having recurrent renal hyperparathyroidism. The indications for tissue aspiration in these 50 lesions were: uncertain or discrepant results between parathyroid scintigraphy and sonography in 47 lesions (94%), unusual locations in 19 lesions (38%), multiple foci in 28 lesions (56%), and locations over previously explored areas in 31 lesions (62%). Eleven patients received two or more tissue aspirate PTH assays for multiple suspicious neck lesions at the same time, and two patients received several tissue aspirate PTH assays at different times. The median serum systemic PTH level before aspiration was 744.25 pg/mL (range 214.78 to >2500.00 pg/mL). Thirty-nine tissue aspirate PTH assays (78%) were positive. After the procedure, no complications were noted after the procedure, such as hemorrhaging, infections, persistent pain, or parathyromatosis.

### 3.2. Comparison between Different Assay Results

The results of the comparison between positive and negative tissue aspirate PTH assays are shown in Table 1. The median tissue aspirate PTH level was 3.9 pg/mL in the assay-negative group and 2500 pg/mL in the assay-positive group (*p* < 0.001). In contrast, there was no significant difference in the levels of serum systemic PTH and calcium between the two groups. A significant correlation was identified between the results of the parathyroid scintigraphy and tissue aspirate PTH assays (*p* = 0.008), whereas no significant correlation was found between the results of parathyroid sonography and tissue aspirate PTH assays (*p* = 0.114).

### 3.3. Comparisons with Localization Studies

Comparisons between the results of tissue aspirate PTH assays and localization studies are shown in Table 2. Among the 50 lesions, three (6%) of them were triple-positive, indicating positive parathyroid scintigraphy, sonography results, and positive tissue aspirate PTH assays. Of the remaining 47 lesions that had uncertain or conflicting localization results between the parathyroid scintigraphy and sonography, 36 were assay-positive (72%) and 11 were assay-negative (22%). 

### 3.4. Surgical Intervention

Thirty-five assay-positive (35/39, 90%) and three assay-negative lesions (3/11, 27%) in 26 patients were explored through neck incisions. All assay-positive and one assay-negative lesions (case 14) were pathologically proven to be parathyroid tissues. Explorations were performed for case 14 because of the uncertainty of the tissue aspiration procedure, which offered borderline assay results. In the other two true assay-negative lesions (case 26-1, 29), removal of the lesions was arranged due to concomitant exploration in the same operation field for other reasons. No surgery was performed for the four assay-positive lesions in the three patients because of presumed severe adhesion from previous management (4 times neck operations and 8 times alcohol injections, case 30) and controllable PTH levels (case 15, 21). The median weight of resected parathyroid glands was 878.0 mg (range 193.0 to 3674.0 mg). In the lesions for which surgery was performed, the positive predictive value of tissue aspirate PTH assays was 100%.

### 3.5. Reoperative Findings

The reoperative findings are shown in Table 3. Among the 36 parathyroid lesions in the 26 patients who underwent surgery, six lesions were ectopic parathyroids, nine lesions were parathyromatosis, and eight lesions were intrathyroid parathyroids. The other 13 lesions were located over the usual parathyroid areas that had already been explored during previous surgical procedures.

## 4. Discussion

In our series, among the patients who received reoperations, 16.7% of the lesions were ectopic, 22.2% were intrathyroid, 36.1% were located over previously explored areas, and 25.0% lesions were confirmed to be parathyromatosis in this study. In challenging medical scenarios, experienced surgeons need comprehensive pre-reoperative localization information to ensure that treatment is achieved. The elevated tissue aspirate PTH assays found with our newly proposed positive assay definitions may help surgeons identify suspicious neck lesions discovered in localization imaging studies with a high positive predictive value (100%) and effectively avoid negative neck explorations in such complicated cases. 

Although tissue aspirate PTH assays can help confirm parathyroid tissue for suspicious neck lesions, not all neck lesions need this examination. We suggest this procedure be reserved for suspicious neck lesions with uncertain or discrepant localization study results, lesions with unusual locations or numbers, lesions over previously explored areas, or a combination of these findings. One of the reasons to use tissue aspirate PTH assays sparingly is the potential for seeding and causing parathyromatosis. To avoid such complications, including seeding along the needle tract, fibrotic tissue reaction, and potential reactive changes of parathyroid lesions [20], the operator should fix the needle and simply oscillate the piston with negative pressure following the needle’s introduction into the center of the suspicious lesion instead of moving the needle tip back and forth, as in other studies [17,19,21]. 

From our series, we found that 71.8% (28/39) of the lesions with positive tissue aspirate PTH assays were considered to be uncertain (suspicious) by parathyroid sonography. Among the positive tissue aspirate PTH assays with uncertain sonographic lesions, 82% (23/28) were scintigraphy-negative or scintigraphy-suspicious lesions. Therefore, tissue aspirate PTH assays may help in identifying lesions that potentially need intervention. Moreover, without the application of tissue aspirate PTH assays, at least nine (23.1%) of the lesions with positive tissue aspirate PTH assays could have been easily missed based on the localization results (negative parathyroid scintigraphy and uncertain parathyroid sonography). 

There is no established standard for the level of PTH that is considered to indicate positive evidence that the aspirated tissue represents parathyroid tissue. Theoretically, the level of PTH in nonparathyroid tissue should be undetectable, as described in a prior study [22]. There have been different positive tissue aspirate PTH values recommended in the individual studies, and the PTH values from different assays are not interchangeable. Frasoldati et al. [21] washed out the tissue aspirates with 1 mL saline and reported a 100% sensitivity and specificity with a cut-off level of 101 pg/mL. Using the same dilution formula, Maser et al. [23] reported that a value higher than the normal range (6–40 pg/mL) indicated parathyroid tissue and a value ranged between 49~65 pg/mL was considered questionable. Stephen et al. [19] diluted a tissue aspirate in 5 mL saline and reported a 94% sensitivity, 77% negative predictive value, and 100% specificity and a positive predictive value when the level was higher than 40 pg/mL. However, by defining the positive tissue aspirate PTH value as higher than the constant or normal PTH level, the results could potentially be contaminated if the blood is aspirated from nonparathyroid tissue and the patient has a high serum PTH [22]. Since the main source of contamination of PTH comes from the aspiration of blood, it is reasonable to define the positive tissue aspirate PTH value through a comparison with the serum PTH. Abdelghani et al. [17] washed out the aspirate with 2 mL saline and reported a 91.6% sensitivity and 100% positive predictive value when the tissue aspirate PTH level was higher than that of serum PTH at the time of performing the aspiration. Nevertheless, the dilution factor of the tissue aspirate to the saline volume was not taken into consideration in Abdelghani et al.’s [17] study and would have produced more false-negative results. In our study, the tissue aspirate was washed out into 6 mL saline, and the volume of aspirate was estimated to be no more than 0.2 mL. Therefore, a positive result was considered when the tissue aspirate PTH level was higher than one-thirtieth (0.2 mL/6 mL) of the serum PTH level. Based on this, the positive predictive value was 100%, and the presumed specificity was 100% (since the possibility of a false-positive result was nearly zero) in our series. Three false-negative results using the criterion of Abdelghani et al. [17] would yield true positive results using our criterion (case 1, 3, 28) (Table S2). 

However, there are limitations to this method. To determine the negative predictive value, sensitivity, and specificity, all the assay-negative lesions should be removed. However, it is important clinically to avoid operations for the potential true negative lesions while managing persistent or recurrent renal hyperparathyroidism. One may argue that our criterion maximizes specificity without providing any evidence for sensitivity; thus, this method may offer a very high positive predictive value but also produce false-negatives. However, based on a comparison with the criteria used in other studies (Table S2), our criterion produces fewer assay-negative lesions under the absence of false positives, which justifies our definition. For the borderline assay-negative lesions (e.g., case 14, 29), repeated tissue aspiration, additional localization studies, or direct explorations should be performed. Our proposed positive assay definition is based on the assumption of the tissue aspirate volume less than 0.2 mL in the needle (0.2 mL/6 mL). Future prospective studies may be warranted to further verify this definition. 

There are several strengths to this study. This is the first and largest series of tissue aspirate PTH assays performed in patients with persistent or recurrent renal hyperparathyroidism. Moreover, this is the first study to describe the complexity of pre-reoperative localization studies with uncertain or discrepant results and how tissue aspirate PTH assays could be helpful in such a difficult medical scenario. Based on the positive value of the tissue aspirate PTH assay, the relative value between the tissue aspirate PTH level and serum PTH level along with the concept of the dilution ratio was proposed to yield ideal results.

## 5. Conclusions

In conclusion, the selective application of a tissue aspirate PTH assay with the newly proposed positive assay definition is feasible and may help clarify the nature of suspicious neck lesions on imaging. This assay may effectively avoid negative neck explorations in patients with complicated persistent or recurrent renal hyperparathyroidism.

## Figures and Tables

**Table 1:** Comparison between cohorts with positive and negative tissue aspirate PTH assays.

Tissue Aspirate PTH Assay	Negative	Positive	*p* Value
Number (*n* = 50)	11	39	
Tissue aspirate PTH level (pg/mL), median (range)	3.9 (1.0–28.6)	2500 (38.9–44470.0)	<0.001
Serum systemic PTH level (pg/mL), median (range)	428.2 (214.8–1817.8)	869.7 (227.8 ≥ 2500.0)	0.137
Serum calcium level (mg/dL), median (range)	10.0 (9.4–12.5)	10.5 (8.8–12.5)	0.218
Parathyroid scintigraphy, *n* (%)			0.008
Negative	9 (81.8%)	13 (33.3%)	
Suspicious	0 (0.0%)	18 (46.2%)	
Positive	2 (18.2%)	8 (20.5%)	
Parathyroid sonography, *n* (%)			0.114
Suspicious	11 (100%)	28 (71.8%)	
Positive	0 (0%)	11 (28.2%)	
Neck lesion explored, *n* (%)	3 (27.3%) ^†^	35 (89.76%) ^‡^	<0.001
Pathology proved parathyroid lesions, *n*	1	35	

^†^ In 3 assay-negative lesions, neck exploration was performed due to borderline assay result (1 pathology proved parathyroid lesion) and concomitant exploration in the same operation field with the other assay-positive lesions. ^‡^ In 4 assay-positive lesions, neck exploration was not performed due to presumed severe adhesion and controllable PTH levels in these patients. Abbreviation: PTH, parathyroid hormone.

**Table 2:** Correlation of the results between tissue aspirate PTH assays and localization studies.

Locolization Studies			Parathyroid Sonography			
			Suspicious (*n* = 39)		Positive (*n* = 11)	
Parathyroid Scintigraphy	Tissue Aspirate PTH Assay		Negative (*n* = 11)	Positive (*n* = 28)	Negative (*n* = 0)	Positive (*n* = 11)
	Negative (*n* = 11) Positive (*n* = 39)					
**Negative** **(*n* = 22)**	Negative (*n* = 9)		9		0	
		Positive (*n* = 13)		9		4
**Suspicious** **(*n* = 18)**	Negative (*n* = 0)		0		0	
		Positive (*n* = 18)		14		4
**Positive** **(*n* = 10)**	Negative (*n* = 2)		2 *		0	
		Positive (*n* = 8)		5		3

* One false-negative tissue aspirate PTH assay included. Abbreviation: PTH, parathyroid hormone.

**Table 3:** Location of parathyroid lesions removed during reoperations.

Location	Lesions (*n* = 36)	Patients (*n* = 26) *
Ectopic		
Undescended	3 (8.3%)	3
Intrathymic	1 (2.8%)	1
Carotid sheath	2 (5.6%)	2
Parathyromatosis	9 (25.0%)	4
Intrathyroid	8 (22.2%)	7
Neck (usual parathyroid area)		
LS	4 (11.1%)	4
LI	1 (2.8%)	1
RS	4 (11.1%)	4
RI	4 (11.1%)	4

* Seven patients had two or more assay-positive lesions removed during the same operation. Abbreviations: LS: left superior; LI: left inferior; RS: right superior; RI: right inferior.

## Data Availability

The data presented in this study are available on request from the corresponding author.

## Supplementary Materials

Supplementary materials can be found at https://www.mdpi.com/2077-0383/10/2/329/s1. Table S1. Demographic data of patients receiving tissue aspirate PTH assays. Table S2. Comparison of tissue aspirate PTH assays results using different criteria.

## Abbreviations

PTH	parathyroid hormone
