# Supplementary material for: Application of Tissue Aspirate Parathyroid Hormone Assay for Imaging Suspicious Neck Lesions in Patients with Complicated Recurrent or Persistent Renal Hyperparathyroidism
Source: J Clin Med. 2021 Jan 18;10(2):329. doi: 10.3390/jcm10020329 (PMC7830567; doi:10.3390/jcm10020329)
Supplement: Supplementary file 1 [file jcm-10-00329-s001.pdf]

Journal of *Clinical Medicine*

MDPI

Application of Tissue Aspirate Parathyroid Hormone Assay for Imaging Suspicious Neck Lesions in Patients with Complicated Recurrent Or Persistent Renal Hyperparathyroidism

Supplementary Material

Table S1. Demographic data of patients receiving tissue aspirate PTH assays.

Case*	Gender (F:M=25:7)	Age (years)	Number of prior operations	Prior operation performed at our hospital (1) or another hospital (2)	Number of parathyroid glands removed during prior operation	Recurrence (R) or Persistence (P) (R:P=8:24)	Lesions underwent tissue aspiration (n=50)	Serum PTH (pg/mL)	Tissue aspirate PTH (pg/mL)	Parathyroidscintigraphy †	Parathyroid sonography†	Reoperative findings (parathyroid location)	Pre-reop PTH (pg/mL)	Post-reop PTH (pg/mL)
1	F	62	1	1	5	R	1	514.61	38.9	n	p	Intravagal	721.77	201.1
2	F	71	1	1	4	P	1	869.7	2150.9	n	p	RS	876.81	215.93
3	F	73	1	1	4	P	2	>2500	>2500	s	s	LS	803.2	302
									712	n	s	intrathyroid		
4	M	46	2	1	6	P	2	624.41	6156.9	p	p	RI	624.41	35.18
									5914.65	n	p	LS		
5	F	66	1	1	4	R	1	412.39	15,807	n	s	undescended	412.39	0.98
6	F	43	1	1	4	P	1	1348	558.53	p	p	RI	1551.4	0.91
7	F	44	1	1	4	P	1	1216	1550	n	s	undescended	954.62	26.14
8	F	60	1	1	3	R	2	428.27	4366	n	s	RI	852.45	90.39
									2.43	n	s	§		
9	M	65	1	1	3	R	1	1404	0.99	n	s	§	1404	52.15
10	F	56	1	1	4	P	1	635	4235.4	p	p	RS	768.92	144.62
11	F	57	1	1	1	R	2	544.74	44470	s	p	RS	1987.6	60.85
									1520.22	s	p	RI		
12	F	53	1	1	4	P	1	832	4132	s	s	undescended	832	4.09
13	F	59	1	1	4	R	2	975.54	10,443.2	s	p	Lt carotid sheath	603.15	155.26
									1.02	n	s	§		
14	M	47	1	1	4	P	1	752.7	18.6	p	s	§ intrathymic	752.71	57.59

15	F	63	2	1	5	R	1	1632.62	519.2	n	s	‡		
16	M	46	2	1	5	P	1	442.16	444.87	s	p	LS	509.2	9.58
17	F	68	1	1	4	P	1	896.7	2591.2	p	s	intrathyroid	2114.2	124.34
18	F	53	1	1	3	P	1	314.6	5.22	n	s	§		
19-1	F	51	1	1	2	P	1	513.47	4967.6	s	s	intrathyroid	963.4	158.46
19-2	F	52	2	1	3	P	1	227.78	822.6	n	s	intrathyroid	789.15	53.88
20	F	51	1	1	4	P	1	524	2744.4	p	s	intrathyroid	936.05	25.85
21	F	63	1	1	4	P	1	297.34	2521.6	N	p	‡		
22	F	55	1	1	4	P	1	748.91	13,934	S	s	intrathyroid	1318.07	111.66
23	F	61	1	1	4	P	1	416.33	1388.16	S	s	intrathyroid	1218.2	27.16
24	F	59	1	2	4	P	2	1675	>2500	S	s	intrathyroid	1079	55.7
									>2500	S	s	parathyromatosis		
25	F	54	4	2	4	R	1	1005	>2500	S	s	LS	1017.91	18.39
26-1	F	54	3	2	5	P	4	1817.8	32,986.5	N	s	parathyromatosis	1377	351
									27,318	N	s	parathyromatosis		
									24,278	N	s	parathyromatosis		
26-2	F	58	4	2	5 + Parathyromatosis	P	3	351	5.14	N	s	§		
									7.4	N	s	§		
									3.93	N	s	§		
									2.21	N	s	§		
27	F	67	1	2	3	P	1	1429.2	3019.3	P	s	LI	769.12	24.03
28	F	51	2	2	Unknown	P	2	998.85	714.44	P	s	parathyromatosis	998.85	307.9
									315.17	P	s	parathyromatosis		
29	M	51	1	2	Unknown	P	2	1417.77	11,296.6	S	s	RS	1417.77	341.72
									28.62	N	s	§		
30	M	52	4	2	Unknown	P	2	739.58	3274	S	s	‡		
									1393	S	s	‡		
31	F	58	1	2	Unknown	P	1	214.7	2.35	P	s	§	1882.8	138.54

								8				
				2			3		>2500	S	s	parathyromatosis
32	M	55	2		Unknown	P		901	>2500	S	s	parathyromatosis
									>2500	S	s	parathyromatosis

*Case 3, 4, 8, 11, 13, 24, 26, 28, 29, 30, 32 received multiple tissue aspirate PTH assays at the same time; Case 19, 26 received multiple tissue aspirate PTH assays at different times; Case 24~32 received prior operations at other hospitals; Case 32 received reoperation at other hospital. †Image study results: p=positive; s=suspicious; n=negative. ‡Assay-positive lesions without operation. §Assay-negative lesions: Case 14 false assay-negative lesion; Case 26-1, 29 received neck operations with assay-negative lesions explored; Case 8, 13 received neck operations without assay-negative lesions explored; Case 9, 31 received graft operations. Abbreviation: PTH, parathyroid hormone; RS, right superior; RI, right inferior; LS, left superior; LI, left inferior.

Table S2. Comparison of tissue aspirate PTH assays results using different criteria.

Case	Study protocol (dilution volume)	Our study (6mL saline)	Frasoldati et al.[19] (1mL saline)	Stephen et al.[18] (5mL saline)	Master et al.[21] (1mL saline)	Abdelghani et al.[16] (2mL saline)
	**Positive assay**	**> serum PTH/30, pg/mL (B*30 > A)**	**> 101 pg/mL (B*6 > 101)**	**> normal serum PTH, pg/mL (B*6/5 > 40 (65))**	**> normal serum PTH, pg/mL (B*6 > 40 (65))**	**> serum PTH, pg/mL (B*3 > A)**
1	**Corrected tissue aspirate PTH, pg/mL**	1167.0	233.4	46.7[+]	233.4	116.7[+]
2		64,527.0	12,905.4	2581.1	12,905.4	6452.7
3		75,000.0	15,000.0	3000.0	15,000.0	7500.0
		21,360.0	4272.0	854.4	4272.0	2136.0[+]
4		184,707.0	36,941.4	7388.3	36,941.4	18,470.7
		177,439.5	35,487.9	7097.6	35,487.9	17,744.0
5		474,210.0	94,842.0	18,968.4	94,842.0	47,421.0
6		16,755.9	3351.2	670.2	3351.2	1675.6
7		46,500.0	9300.0	1860.0	9300.0	4650.0
8		130,980.0	26,196.0	5239.2	26,196.0	13,098.0
		72.9[*]	14.6[*]	2.9[*]	14.6[*]	7.3[*]
9		29.7[*]	5.9[*]	1.2[*]	5.9[*]	3.0[*]
10		127,062.0	25,412.4	5082.5	25,412.4	12,706.2
11		1,334,100.0	266,820.0	53,364.0	266,820.0	133,410.0
		45,606.6	9121.3	1824.3	9121.3	4560.7
12		123,960.0	24,792.0	4958.4	24,792.0	12,396.0
13		313,296.0	62,659.2	12,531.8	62,659.2	31,329.6
		30.6[*]	6.1[*]	1.2[*]	6.1[*]	3.1[*]
14		558.0[+]	111.6	22.3[+]	111.6	55.8[+]
15		15,576.0	3115.2	623.0	3115.2	1557.6
16		13,346.1	2669.2	533.8	2669.2	1334.6
17		77,736.0	15,547.2	3109.4	15,547.2	7773.6
18		156.6[*]	31.3[*]	6.3[*]	31.3[*]	15.7[*]
19-1		149,028.0	29,805.6	5961.1	29,805.6	14,902.8
19-2		24,678.0	4935.6	987.1	4935.6	2467.8
20		82,332.0	16,466.4	3293.3	16,466.4	8233.2
21		75,648.0	15,129.6	3025.9	15,129.6	7564.8
22		418,020.0	83,604.0	16,720.8	83,604.0	41,802.0
23		41,644.8	8329.0	1665.8	8329.0	4164.5
24		75,000.0	15,000.0	3000.0	15,000.0	7500.0
		75,000.0	15,000.0	3000.0	15,000.0	7500.0
25		75,000.0	15,000.0	3000.0	15,000.0	7500.0
		989,595.0	197,919.0	39,583.8	197,919.0	98,959.5
26-1		819,540.0	163,908.0	32,781.6	163,908.0	81,954.0
		728,340.0	145,668.0	29,133.6	145,668.0	72,834.0

	154.2^{*}	30.8^{*}	6.2^{*}	30.8^{*}	15.4^{*}
	222.0^{*}	44.4^{*}	8.9^{*}	44.4^{*}	22.2^{*}
26-2	117.9^{*}	23.6^{*}	4.7^{*}	23.6^{*}	11.8^{*}
	66.3^{*}	13.3^{*}	2.7^{*}	13.3^{*}	6.6^{*}
27	90,579.0	18,115.8	3623.2	18,115.8	9057.9
28	21,433.2	4286.6	857.3	4286.6	2143.3
	9455.1	1891.0	378.2	1891.0	945.5^{+}
29	338,898.0	67,779.6	13555.9	67,779.6	33,889.8
	858.6^{*}	$171.7^{\#}$	34.3^{*}	$171.7^{\#}$	85.9^{*}
30	98220.0	19,644.0	3928.8	19,644.0	9822.0
	41790.0	8358.0	1671.6	8358.0	4179.0
31	70.5^{*}	14.1^{*}	2.8^{*}	14.1^{*}	7.1^{*}
	75,000.0	15,000.0	3000.0	15,000.0	7500.0
32	75,000.0	15,000.0	3000.0	15,000.0	7500.0
	75,000.0	15,000.0	3000.0	15,000.0	7500.0

*Assay-negative result (according to the individual criteria). †False assay-negative result (according to the individual criteria). #False assay-positive result (according to the individual criteria). A, Serum PTH (pg/mL) in Supplementary Table S1; B, Tissue aspirate PTH (pg/mL) in Supplementary Table S1. Abbreviation: PTH, parathyroid hormone.

© 2020 by the authors. Licensee MDPI, Basel, Switzerland. This article is an open access article distributed under the terms and conditions of the Creative Commons Attribution (CC BY) license (http://creativecommons.org/licenses/by/4.0/).
